# Safety of a Tailored Gadolinium-Based Contrast Agent Protocol Considering Excretion Pathways in Patients with Renal Impairment

**DOI:** 10.3390/diagnostics16030451

**Published:** 2026-02-01

**Authors:** Jeong Woo Kim, Chang Hee Lee, Gang-Jee Ko, Sang-Il Suh

**Affiliations:** 1 Department of Radiology, Korea University Guro Hospital, Korea University College of Medicine, 148 Gurodong-ro, Guro-gu, Seoul 08380, Republic of Korea; pridebio@naver.com (J.W.K.);; 2 Department of Internal Medcine, Korea University Guro Hospital, Korea University College of Medicine, Seoul 08308, Republic of Korea

**Keywords:** contrast media, gadolinium-based contrast agent, nephrogenic systemic fibrosis, safety

## Abstract

**Background/Objectives:** Considering the excretion pathways and administered gadolinium dose, our institution has developed a tailored gadolinium-based contrast agents (GBCAs) administration protocol for patients with renal impairment to facilitate more rapid elimination and minimal retention of gadolinium. This study aims to evaluate the 8-year clinical outcomes and safety of this institutional protocol. **Methods:** This single-center retrospective study included patients with renal impairment who underwent GBCA-enhanced MRI between January 2015 and December 2022. The protocol recommended specific GBCAs and adjusted doses based on chronic kidney disease (CKD) stage and serum bilirubin levels: gadoxetate disodium was used for normal serum bilirubin level due to its dual excretion pathway, while macrocyclic agents were used for those with elevated serum bilirubin levels. During the follow-up period, occurrence of nephrogenic systemic fibrosis (NSF) and evidence of gadolinium deposition in brain tissues were evaluated. **Results:** A total of 288 patients (age, 64.6 ± 11.7 years; male, 64.9%) underwent 716 GBCA-enhanced MRI examinations in accordance with the institutional protocol. The cohort included 62 patients with CKD stage 4 and 131 patients with CKD stage 5 or undergoing hemodialysis. In patients with CKD stage 4 and 5 and those undergoing hemodialysis, 597 examinations were performed using gadoxetate disodium, and 119 used macrocyclic agents. No cases of NSF or gadolinium deposition in brain tissues were identified over mean follow-up intervals of 27.5 and 27.8 months, respectively. **Conclusions:** The tailored GBCA administration protocol, considering the excretion pathways and administered gadolinium dose, appears to be safe with respect to NSF for patients with renal impairment, and no evidence of brain gadolinium deposition was observed in the evaluated subset of patients.

## 1. Introduction

Although gadolinium-based contrast agents (GBCAs) have been widely used in contrast-enhanced MRI examinations, safety concerns—particularly related to nephrogenic systemic fibrosis (NSF)—have been raised regarding their use in patients with renal impairment [1]. Various GBCAs differ in chelate stability, molecular structure, and physicochemical properties, which affect gadolinium retention and excretion from the body [2]. Current clinical practice and international guidelines preferentially recommend macrocyclic GBCAs due to their higher kinetic stability and low risk of NSF [3]. Although gadoxetate disodium, a more recently introduced linear ionic GBCA, is classified as a group III (likely very low risk but insufficient confirmatory evidence) by the American College of Radiology (ACR), several recent studies have reported that gadoxetate disodium is safe for NSF in patients with renal impairment [4,5,6].

If gadolinium is not adequately excreted and consequently retained in the body, it may accumulate in tissues and lead to serious adverse effects. In patients with renal impairment, prolonged gadolinium retention has been associated with NSF, a rare but severe and potentially life-threatening systemic disorder characterized by progressive fibrosis involving the skin and internal organs [2]. Impaired renal clearance may increase the likelihood of dissociation of free Gd3+ from its chelate, which is considered a key mechanism underlying the development of NSF. Therefore, it is important to rapidly eliminate gadolinium from the body and minimize its retention in these patients. Separately, gadolinium deposition in the brain, typically manifesting as T1 hyperintensity in the globus pallidus and dentate nucleus on unenhanced MRI, has been reported following repeated GBCA administration. This phenomenon involves substantially lower amounts of retained gadolinium than those associated with NSF, and its clinical consequences remain uncertain, with no definitive neurologic toxicity demonstrated to date [7].

Most GBCAs are eliminated through renal excretion via glomerular filtration, but some GBCAs have a dual excretion pathway in which they are also excreted through the hepatobiliary system [3]. Gadoxetate disodium is a hepatocyte-specific GBCA, approximately 50% of which is taken up by functioning hepatocytes and excreted into bile [3,8]. Previous experimental and clinical studies have demonstrated that these dual excretion pathways may facilitate compensatory elimination in the setting of renal dysfunction, potentially reducing gadolinium retention in patients with impaired renal function [9,10,11]. Additionally, because the gadolinium concentration of gadoxetate disodium is lower (0.25 M) than that of other GBCAs (0.5 or 1.0 M), the total molar dose of gadolinium administered to the patient (mmol/kg) is lower when the same volume is injected.

Our institution developed a tailored GBCA administration protocol that considers excretion pathways and administered gadolinium dose for patients with renal impairment. The institutional protocol was designed to use macrocyclic agents as the standard choice in patients with elevated serum bilirubin levels, while selectively incorporating gadoxetate disodium in patients with normal serum bilirubin levels, taking advantage of its dual excretion pathway and lower administered gadolinium dose.

The purpose of this study was to evaluate the long-term safety and clinical outcomes of this institutional protocol. We investigated whether this tailored approach, implemented over an 8-year period, effectively prevents the occurrence of NSF and gadolinium deposition in brain tissue in patients with renal impairment.

## 2. Materials and Methods

### 2.1. Patients

**Figure 1:**
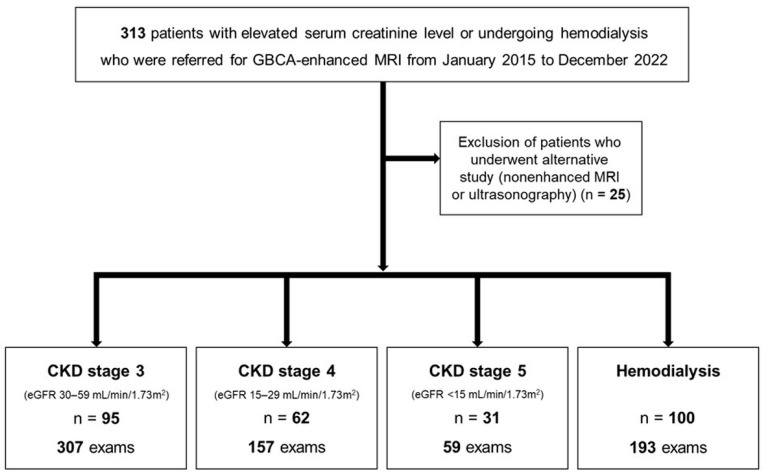
Flow diagram of the study population.

This retrospective study was approved by our institutional review board, and the requirement for informed consent was waived. Since 2015, all GBCA-enhanced MRI examinations at our institution have been performed in accordance with a tailored GBCA administration protocol developed for patients with renal impairment or undergoing hemodialysis. Patients with elevated serum creatinine or undergoing hemodialysis who were referred for GBCA-enhanced MRI were consecutively recruited from January 2015 to December 2022 (Figure 1).

**Figure 2:**
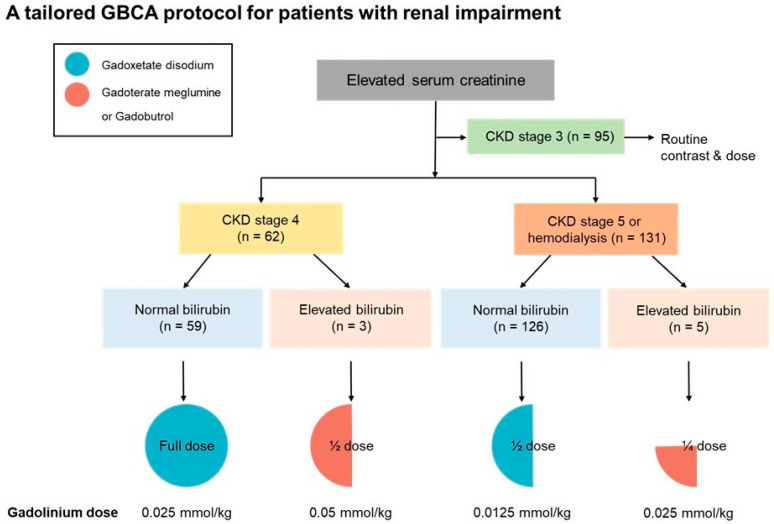
A tailored gadolinium-based contrast agent (GBCA) protocol considering excretion pathways and administered gadolinium dose in patients with renal impairment. The institutional protocol recommends specific GBCAs at an adjusted dose based on chronic kidney disease (CKD) stage and serum bilirubin levels. If the serum bilirubin level is elevated (>1.2 mg/dL), macrocyclic agent is recommended. If the serum bilirubin level is within the normal range (≤1.2 mg/dL), gadoxetate disodium is recommended to utilize its dual excretion pathways.

**Figure 3:**
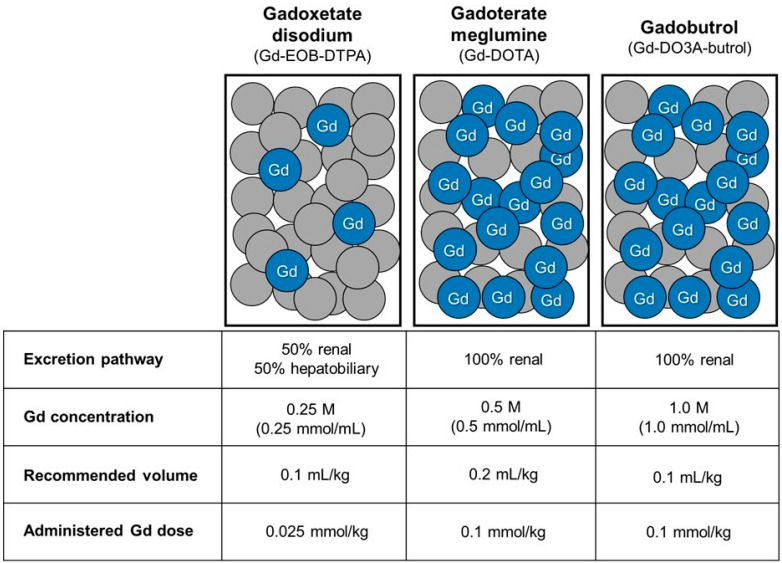
Excretion pathway and administered gadolinium dose of gadolinium-based contrast agents (GBCAs). GBCAs differ in their excretion pathways and the amount of gadolinium administered per body weight. The number of blue spheres represents the relative administered gadolinium dose (mmol/kg) for each agent.

The institutional protocol recommended specific GBCAs at an adjusted dose based on the chronic kidney disease (CKD) stage and serum bilirubin levels. For patients with CKD stage 3, full-dose GBCAs were administered as in patients with normal renal function. For patients with CKD stage 4, a full dose of gadoxetate disodium was recommended in patients with normal serum bilirubin levels, whereas a half-dose of gadoterate meglumine or gadobutrol was administered in patients with elevated serum bilirubin levels. For patients with CKD stage 5 or undergoing hemodialysis, a half-dose of gadoxetate disodium was recommended in patients with normal serum bilirubin levels, while a quarter-dose of gadoterate meglumine or gadobutrol was used in those with elevated serum bilirubin levels (Figure 2). Because gadoxetate disodium has a lower molar concentration (0.25 M) than macrocyclic GBCAs, including gadoterate meglumine (0.5 M) and gadobutrol (1.0 M), a relatively higher volumetric dose of gadoxetate disodium was administered in patients with normal bilirubin levels; however, the total administered gadolinium dose remained lower (Figure 2 and Figure 3). Serum total bilirubin levels were used to stratify patients. Normal bilirubin was defined as ≤1.2 mg/dL, and elevated bilirubin as >1.2 mg/dL, based on the institutional upper limit of normal and commonly accepted clinical reference ranges.

All patients underwent serum creatinine and bilirubin testing within three days prior to MRI examination. The estimated glomerular filtration rate (eGFR) was calculated using the Modification of Diet in Renal Disease study equation using the serum creatinine level [12]. CKD stages were defined as follows: stage 1, eGFR > 90 mL/min/1.73 m^2^; stage 2, eGFR 60–89 mL/min/1.73 m^2^; stage 3, eGFR 30–59 mL/min/1.73 m^2^; stage 4, eGFR 15–29 mL/min/1.73 m^2^; and stage 5, eGFR < 15 mL/min/1.73 m^2^. Because eGFR is not a reliable indicator of renal function in patients undergoing hemodialysis, eGFR was not used for staging in these patients. The follow-up period was determined as the interval between the date of the GBCA-enhanced MRI examination and the date of the last outpatient visit or hospitalization.

### 2.2. Clinical Outcomes

To identify NSF cases, we conducted a comprehensive review of the patients’ electronic medical records. The diagnosis of NSF was based on the clinicopathological scoring system outlined by Girardi et al. [13]. Patients were considered positive for NSF only if evaluated by a board-certified dermatologist and confirmed by deep skin biopsy. Cases of NSF mentioned only as a differential diagnosis or probable diagnosis without histological confirmation were excluded.

Gadolinium deposition in brain tissues was evaluated in patients who (1) underwent brain MRI examinations both before and after GBCA-enhanced MRI or (2) underwent brain MRI as the initial GBCA-enhanced study and had subsequent follow-up brain MRIs. T1 hyperintensity in the dentate nucleus and globus pallidus was evaluated on pre-contrast images. Because T1 hyperintensity in these regions may be observed in various other clinical conditions [7], cases in which only post-GBCA brain MRI was available or gadolinium deposition could not be reliably identified were excluded from the brain deposition analysis.

### 2.3. Image Quality Analysis for Non-Hepatobiliary MRI Examinations

To assess the image quality of non-hepatobiliary MRI examinations, a comparative analysis was performed between two groups. The first group consisted of non-hepatobliary MRI examinations enhanced with gadoxetate disodium in patients with CKD stage 4, 5, and undergoing hemodialysis. The second group included non-hepatobiliary MRI examinations performed in patients with CKD stage 3 using standard full-dose macrocyclic GBCAs.

All included examinations from both groups were pooled and independently evaluated by two board-certified radiologists, who were blinded to clinical information and contrast agent type. Image quality was assessed using a 5-point Likert scale, with higher scores indicating better diagnostic adequacy, for (1) overall diagnostic adequacy, (2) contrast enhancement adequacy, and (3) lesion conspicuity, when a lesion was present. In addition, all examinations were reviewed to identify cases requiring repeat MRI or alternative imaging modalities due to insufficient enhancement or non-diagnostic image quality. Discrepancies were resolved by consensus.

### 2.4. Statistical Analysis

Continuous variables are presented as mean ± standard deviation with range, and categorical variables are presented as frequency and percentage. The risk of NSF in patients with renal impairment was calculated per patient and examination, with the upper bound of 95% confidence interval (CI) determined using the Wilson score without continuity correction [14].

For comparison of image quality between non-hepatobiliary MRI examinations enhanced with gadoxetate disodium and those performed with standard full-dose macrocyclic GBCAs, Likert-scale image quality scores were compared using the Mann–Whitney U test. The proportion of examinations requiring repeat MRI or alternative imaging modalities was compared descriptively between groups. A two-sided *p*-value < 0.05 was considered statistically significant. All statistical analyses were performed using SPSS (version 26.0; IBM Corp., Armonk, NY, USA).

## 3. Results

### 3.1. Patients

A total of 313 patients were referred for GBCA-enhanced MRI examinations. Among them, 288 patients underwent 716 GBCA-enhanced MRI examinations in accordance with our institutional protocol, while 25 patients underwent alternative imaging studies, including nonenhanced MRI or ultrasonography (Figure 1). The mean age of the 288 patients at the time of GBCA-enhanced MRI was 64.6 ± 11.7 years (range, 29–93 years), and 187 (64.9%) were men. Baseline demographic characteristics of the patients are summarized in Table 1. The cohort included 95 patients with CKD stage 3, 62 with CKD stage 4, 31 with CKD stage 5, and 100 patients undergoing hemodialysis (Figure 1). Among the 288 patients, 149 (51.7%) patients underwent more than one GBCA-enhanced MRI examination in accordance with the tailored GBCA administration protocol during the study period (Table 2). Sixty patients (20.8%) had undergone GBCA-enhanced MRI examinations prior to the implementation of this protocol. During the study period, the mean cumulative gadolinium dose was 0.070 ± 0.093 mmol/kg (range, 0.0125–0.9 mmol/kg) for the entire cohort and 0.025 ± 0.023 mmol/kg (range, 0.0125–0.1625 mmol/kg) specifically for patients with CKD5 or undergoing hemodialysis.

In patients with CKD stage 4, 152 full-dose gadoxetate disodium-enhanced MRI examinations and 5 half-dose gadoterate meglumine or gadobutrol-enhanced MRI examinations were performed. In patients with CKD stage 5 or those undergoing hemodialysis, 242 half-dose gadoxetate disodium-enhanced MRI examinations and 10 quarter-dose gadoterate meglumine or gadobutrol-enhanced MRI examinations were performed. Table 3 summarizes the distribution of GBCAs with adjusted doses based on renal function and serum bilirubin levels across different anatomical regions.

The mean follow-up period for all GBCA-enhanced MRI examinations was 27.5 ± 31.0 months (range, 1–120 months) (Table 1).

### 3.2. Nephrogenic Systemic Fibrosis (NSF)

No cases of NSF were identified among patients with renal impairment who underwent MRI following our institutional protocol. Consequently, the overall risk of NSF in this population was 0% (95% CI, 0–1.32%). Among patients with CKD stages 4, 5, and undergoing hemodialysis, the upper bound of 95% CI was 1.95% per patient (n = 193) and 0.93% per examination (n = 409).

### 3.3. Gadolinium Deposition in Brain Tissue

Assessment of gadolinium deposition in brain tissue was performed in 26 patients (15 with CKD stage 5 or undergoing hemodialysis, 4 with CKD stage 4, and 7 with CKD stage 3) who had available pre- and post-GBCA brain MRI or follow-up imaging. These patients underwent an average of 3.9 ± 1.8 GBCA-enhanced MRI examinations, and the mean interval between the GBCA-enhanced MRI and the follow-up brain MRI was 27.8 ± 17.1 months (range, 12–61 months). Compared with the remaining cohort, patients included in the brain MRI analysis underwent a significantly higher number of GBCA-enhanced MRI examinations (3.9 ± 1.8 vs. 2.5 ± 2.3 examinations per patient, *p* < 0.001) and had a significantly higher cumulative gadolinium dose (0.097 ± 0.086 vs. 0.070 ± 0.092 mmol/kg, *p* = 0.002). Despite this higher gadolinium exposure, no cases demonstrated new T1 hyperintensity in the globus pallidus or dentate nucleus on follow-up brain MRI.

### 3.4. Image Quality Analysis for Non-Hepatobiliary MRI Examinations

A total of 239 non-hepatobiliary MRI examinations enhanced with gadoxetate disodium in patients with CKD stage 4, 5, and undergoing hemodialysis and 125 examinations performed in patients with CKD stage 3 using full-dose macrocyclic GBCAs were included in the image quality analysis. Mean Likert scores for overall diagnostic adequacy (4.70 ± 0.56 vs. 4.74 ± 0.53; *p* = 0.55), contrast enhancement adequacy (4.81 ± 0.49 vs. 4.85 ± 0.40; *p* = 0.71), and lesion conspicuity (3.74 ± 2.02 vs. 3.76 ± 2.02; *p* = 0.90) demonstrated no statistically significant differences between groups. Notably, no examinations in either group received Likert scores of 1 or 2 for each category.

In addition, no examinations in either group required repeat MRI or alternative imaging modalities due to insufficient enhancement or non-diagnostic image quality (0% in both groups).

## 4. Discussion

The present study evaluated the long-term clinical safety of a tailored GBCA administration protocol that considers excretion pathways and administered gadolinium dose in patients with renal impairment. Over an 8-year period, no cases of NSF were identified among 288 patients with renal impairment, including 131 patients with CKD stage 5 or undergoing hemodialysis. In addition, in the subset of patients who underwent brain MRI, no apparent signal changes suggestive of gadolinium deposition were observed. Notably, this finding was observed despite higher cumulative gadolinium dose and a greater number of GBCA-enhanced MRI examinations compared with the remaining cohort; however, this finding should be interpreted cautiously given the limited sample size. Although image quality was not a primary outcome of this study, the additional analysis of non-hepatobiliary MRI examinations demonstrated preserved diagnostic adequacy with gadoxetate disodium, comparable to standard full-dose macrocyclic agents, without the need for repeat or alternative imaging.

GBCAs are administered in a chelated form to reduce the toxicity of free gadolinium ions [15]. The stability of the chelate and pharmacokinetic properties influences gadolinium retention and elimination. In patients with normal renal function, most administered gadolinium is rapidly eliminated. However, in patients with renal impairment, reduced clearance may result in prolonged retention and increased susceptibility to tissue deposition [16,17]. Therefore, strategies that facilitate alternative excretion pathways and minimize total gadolinium exposure are particularly important in this patient population. Our GBCA administration protocol was designed to address these concerns by tailoring GBCA and dosing based on renal function and serum bilirubin levels.

Although most GBCAs are eliminated through renal excretion, certain agents, including gadoxetate disodium and gadobenate dimeglumine, exhibit an additional hepatobiliary excretion pathway [8]. The physiological significance of this alternative pathway becomes particularly evident when renal function is compromised. Experimental studies using animal models with impaired renal or hepatic function have demonstrated compensatory excretion of these agents through alternative pathways, resulting in more rapid elimination compared with purely renally excreted GBCAs. Mühler et al. reported that in animals with renal vessel ligation, gadoxetate disodium showed significantly greater elimination and lower retention 8 h after injection compared with gadopentetate dimeglumine (1.3% vs. 96.3% retention) [9]. Similarly, Kirchin et al. confirmed that biliary excretion increases proportionally when renal pathways are occluded [10]. Complementing these experimental observations, a clinical pharmacokinetic study by Gschwend et al. have shown that hepatobiliary excretion of gadoxetate disodium may partially compensate for impaired renal clearance in patients with mild to moderate renal impairment [11]. Collectively, based on these findings, we developed our institutional protocol to prioritize these dual excretion pathway agents according to the serum bilirubin levels in patients with renal impairment. By strategically utilizing the hepatobiliary excretion pathway, this tailored GBCA administration protocol aims to facilitate gadolinium elimination and minimize systemic retention in patients with renal impairment.

Although gadoxetate disodium is categorized as a group III agent (likely very low risk but with limited confirmatory evidence) by the ACR and as an intermediate-risk agent by the European Society of Urogenital Radiology (ESUR), clinical evidence regarding its safety in patients with renal impairment is steadily accumulating [4,5,6,18,19,20,21,22]. Lauenstein et al. found no NSF cases in a prospective study of 278 patients with moderate-to-severe renal impairment [18], and Starekova et al. demonstrated similar safety outcomes even with double-dose administrations in 183 patients with severe renal impairment [4]. The absence of NSF observed in the present study is consistent with these previous findings and further supports the accumulating data on the safety of gadoxetate disodium in patients with renal impairment. The present study differs from previous studies in that it evaluated a tailored GBCA administration protocol considering excretion pathways and administered gadolinium dose.

Although our study lacks a direct control group, the absence of NSF observed in our cohort should be interpreted in the context of accumulating evidence from large published cohorts and meta-analysis involving patients with renal impairment. In particular, a comprehensive systematic review and meta-analysis by Woolen et al. evaluated 16 studies comprising 4931 patients with stage 4 or 5 chronic kidney disease who received group II GBCAs, including macrocyclic agents and gadobenate dimeglumine, and reported a pooled NSF incidence of 0%, with an upper bound of the 95% CI of only 0.07% [23]. Several large observational studies focusing specifically on gadoxetate disodium administration in patients with renal impairment have also reported no cases of NSF [4,5,6]. In a cohort of more than 7800 gadoxetate disodium-enhanced MRI examinations, including patients with eGFR < 30 mL/min/1.73 m^2^ and those undergoing dialysis, Starekova et al. reported no cases of NSF, with upper bound of the 95% CI of approximately 2.1% [4]. Similar findings were reported by Tseng et al., who observed no NSF cases among patients with moderate-to-severe renal impairment receiving fixed-dose gadoxetate disodium, despite repeated administrations and cumulative dosing, yielding an upper 95% CI limit of 2.2% [5]. More recently, Gauthier et al. combined institutional data with an updated literature review and reported zero cases of NSF among 340 patients with moderate or severe renal impairment exposed to gadoxetate disodium, corresponding to an upper 95% CI of 0.9% [6]. Taken together, these data indicate that the safety profile observed in our study aligns closely with contemporary evidence and supports the use of a tailored GBCA protocol that accounts for alternative excretion pathways and administered gadolinium dose in patients with renal impairment.

Another safety concern associated with GBCA administration is gadolinium deposition in brain tissues. Since the initial report by Kanda et al. [24], several studies have demonstrated an association between repeated GBCA administration and increased T1 hyperintensity in the dentate nucleus and globus pallidus. These findings have been consistently reported; however, their clinical significance remains unclear, and no clear neurological and cognitive sequelae have been identified to date [7].

There have been several limitations in this study. First, this study was a retrospective study, with no direct comparison with a control group receiving GBCAs without dose adjustment. However, when our institutional protocol was developed 10 years ago, it would have raised ethical concerns, given limited safety evidence at the time. In addition, the primary aim of this study was not comparative efficacy but the evaluation of long-term safety outcomes following implementation of a tailored GBCA administration protocol in the actual clinical setting. Furthermore, as this study was conducted at a single institution with a highly structured protocol and experienced radiology staff, the generalizability of the findings to other clinical settings may be limited and requires further validation in multicenter studies. Second, the number of patients included the assessment of gadolinium deposition in brain tissue was relatively small. Although patients included in the brain MRI analysis underwent a greater number of GBCA-enhanced MRI examinations and had higher cumulative gadolinium doses than the remaining cohort, the limited sample size warrants cautious interpretation of the absence of detectable brain signal changes and restricts the generalizability of these findings. Future prospective studies with large cohorts are warranted to further validate the long-term neurobiological safety of this tailored protocol. Third, although gadoxetate disodium is primarily indicated as a hepatobiliary-specific agent, its use for non-hepatobiliary imaging—including brain and musculoskeletal MRI—represents off-label application within our institutional protocol to prioritize patient safety in patients with renal impairment. In our study, supplementary image quality analyses demonstrated preserved diagnostic adequacy, with no examinations receiving low Likert scores for diagnostic or enhancement adequacy and no cases requiring repeat or alternative imaging, when compared with full-dose macrocyclic agents. Meanwhile, the clinical feasibility of adjusted GBCA dose in patients with renal impairment has been demonstrated in previous studies [21,25]. In particular, Song et al. demonstrated that half-dose gadoxetate disodium-enhanced liver MRI maintained adequate diagnostic performance in patients at risk for NSF [21]. Similarly, de Campos et al. reported that a quarter-dose (0.025 mmol/kg) of the dual-excretion agent gadobenate dimeglumine could be used for abdominal MRI in patients with renal impairment without compromising diagnostic image quality [25]. Consistent with these prior observations, the supplementary image quality analyses in the present study suggest that selective dose-adjusted use of gadoxetate disodium may be clinically acceptable in carefully selected non-hepatobiliary MRI examinations. Finally, delayed onset of NSF has been reported 10 years after GBCA exposure [26,27], so cases NSF with delayed onset may have been missed; however, such occurrences considered very rare, and the relatively long follow-up period of this study provides relatively high confidence in safety.

## 5. Conclusions

In conclusion, this study demonstrated that a tailored GBCA administration protocol can be associated with favorable long-term safety outcomes in patients with renal impairment. Over an 8-year follow-up period, no cases of NSF were observed, supporting the safety of this protocol with respect to this severe systemic complication. Separately, no evidence of brain gadolinium deposition was identified in the evaluated subset of patients; however, this finding should be interpreted with caution given the limited sample size. Therefore, our institutional GBCA administration protocol, which strategically considers excretion pathways and administered gadolinium dose to facilitate rapid elimination and minimize retention of gadolinium, may contribute to safer clinical management of patients with renal impairment.

## Figures and Tables

**Table 1:** Baseline patient characteristics.

Variables	CKD 3 (*n* = 95)	CKD 4 (*n* = 62)	CKD 5 (*n* = 31)	Hemodialysis (*n* = 100)	Overall (*n* = 288)
Male, n (%)	73 (76.8)	41 (66.1)	15 (48.4)	58 (58.0)	187 (64.9)
Age (years)	66.7 ± 11.4	68.0 ± 11.0	62.0 ± 12.1	63.2 ± 12.0	64.6 ± 11.7
Follow-up period (months)	26.2 ± 30.8 (range 1–120)	34.7 ± 31.3 (range 1–120)	27.8 ± 37.4 (range 1–120)	24.1 ± 28.8 (range 1–120)	27.5 ± 31.0 (range 1–120)

Note.—Continuous data (age and follow-up period) are presented as mean ± standard deviation and categorical data are presented as numbers (%). CKD = chronic kidney disease.

**Table 2:** Distribution of repeated GBCA-enhanced MRI examinations according to renal function.

No. of GBCA-Enhanced MRI Examination	CKD 3 (*n* = 95)	CKD 4 (*n* = 62)	CKD 5 (*n* = 31)	Hemodialysis (*n* = 100)
1 exam	30	32	19	55
2 exams	22	11	6	25
3 exams	10	9	2	10
≥4 exams	33	10	4	10

Note.—GBCA = gadolinium-based contrast agent, CKD = chronic kidney disease.

**Table 3:** Distribution of GBCAs with adjusted doses based on renal function and serum bilirubin levels across anatomical regions.

Renal Function	CKD 4		CKD 5 or Hemodialysis	
Serum Bilirubin Levels	Normal ^†^	Elevated ^‡^	Normal ^§^	Elevated *^∥^*
Abdomen	88	2	67	5
Neuro (Brain/Spine)	46	1	109	4
MSK, Others (Chest, Breast)	18	2	66	1
Total	152	5	242	10

Note.—Data are the number of GBCA-enhanced MRI examinations. CKD = chronic kidney disease, GBCA = gadolinium-based contrast agent, MSK = musculoskeletal. ^†^ Full dose gadoxetate disodium was administered. ^‡^ Half-dose macrocyclic agent (either gadoterate meglumine or gadobutrol) was administered. ^§^ Half-dose gadoxetate disodium was administered. *^∥^* Quarter-dose macrocyclic agent (either gadoterate meglumine or gadobutrol) was administered.

## Data Availability

The data presented in this study are available on request from the corresponding author. The data are not publicly available due to institutional privacy and ethical restrictions.

## Abbreviations

The following abbreviations are used in this manuscript:

GBCA	Gadolinium-based contrast agent
NSF	Nephrogenic systemic fibrosis
ACR	American college of radiology
CKD	Chronic kidney disease
eGFR	Estimated glomerular filtration rate
CI	Confidence interval
ESUR	European society of urogenital radiology
